# Different Roles of Eukaryotic MutS and MutL Complexes in Repair of Small Insertion and Deletion Loops in Yeast

**DOI:** 10.1371/journal.pgen.1003920

**Published:** 2013-10-31

**Authors:** Nina V. Romanova, Gray F. Crouse

**Affiliations:** 1 Department of Biology, Emory University, Atlanta, Georgia, United States of America; 2 Winship Cancer Institute, Emory University, Atlanta, Georgia, United States of America; Duke University, United States of America

## Abstract

DNA mismatch repair greatly increases genome fidelity by recognizing and removing replication errors. In order to understand how this fidelity is maintained, it is important to uncover the relative specificities of the different components of mismatch repair. There are two major mispair recognition complexes in eukaryotes that are homologues of bacterial MutS proteins, MutSα and MutSβ, with MutSα recognizing base-base mismatches and small loop mispairs and MutSβ recognizing larger loop mispairs. Upon recognition of a mispair, the MutS complexes then interact with homologues of the bacterial MutL protein. Loops formed on the primer strand during replication lead to insertion mutations, whereas loops on the template strand lead to deletions. We show here in yeast, using oligonucleotide transformation, that MutSα has a strong bias toward repair of insertion loops, while MutSβ has an even stronger bias toward repair of deletion loops. Our results suggest that this bias in repair is due to the different interactions of the MutS complexes with the MutL complexes. Two mutants of MutLα, *pms1-G882E* and *pms1-H888R*, repair deletion mispairs but not insertion mispairs. Moreover, we find that a different MutL complex, MutLγ, is extremely important, but not sufficient, for deletion repair in the presence of either MutLα mutation. MutSβ is present in many eukaryotic organisms, but not in prokaryotes. We suggest that the biased repair of deletion mispairs may reflect a critical eukaryotic function of MutSβ in mismatch repair.

## Author Summary

DNA mismatch repair is a major pathway that prevents both base substitution and insertion or deletion errors during replication. Most eukaryotes have two recognition complexes, MutSα and MutSβ, homologues of prokaryotic MutS and differing in their affinity for mismatches, with MutSα recognizing base-base mismatches and small insertion/deletion loops and MutSβ recognizing larger loops. We show that repair mediated by these complexes has opposite biases for insertion versus deletion mispairs with MutSα-directed repair favoring insertion loops and MutSβ-directed repair favoring deletion loops. This bias is mediated by differing interactions with downstream MutL complexes. We suggest that MutSα represents a prokaryotic MutS biased for repair of insertion loops and that MutSβ represents a new eukaryotic activity biased for repair of deletion loops.

## Introduction

DNA mismatch repair (MMR) is a major repair system in organisms ranging from bacteria to humans. The discovery that MMR defects cause the most common form of inherited colon cancer underscored the importance of this repair pathway to human health [1]–[6]. In eukaryotes, MMR involves recognition of mismatches created during replication by protein complexes that are homologues of bacterial MutS, followed by downstream processing events involving homologues of bacterial MutL [7]–[9]. There are two main recognition complexes, MutSα, a heterodimer consisting of Msh2 and Msh6 that recognizes base-base mismatches and small loops, and MutSβ, a heterodimer consisting of Msh2 and Msh3 that recognizes mainly loops [7]–[10].

The exact role that MutSβ plays in MMR is not clear. Loss of MutSβ causes only a weak mutator effect unless the assay is specific for insertion or deletion (in/del) mutations [11], [12]. In general, there seems to be much less MutSβ protein than MutSα protein in yeast and human cells [13]–[15]; however, a recent report suggests that the relative amounts of MutSα and MutSβ vary in mouse tissues, with some tissues containing more MutSβ than MutSα [16]. A number of organisms such as *Drosophila melanogaster* and *Caenorhabditis elegans* apparently have no MutSβ although they have MutSα [17]. Most analysis of MutSβ MMR function has tended to center on its repair of loops compared to the repair of base-base mismatches by MutSα. However, two early studies of MutSβ and microsatellite instability in yeast found a surprising difference in loop repair and loss of MutSβ compared to loss of MutSα [18], [19]. For example, using an assay for dinucleotide repeat slippage, Sia *et al.* found more insertions than deletions in wild-type cells, whereas complete loss of MMR resulted in approximately equal numbers of insertions and deletions; strikingly, cells containing only MutSα had many more deletions than insertions whereas cells containing only MutSβ had many more insertions than deletions [18]. The authors concluded that loops on the primer strand were repaired differently from loops on the template strand.

The role of MutL proteins in MMR is less well understood, although they act downstream of initial mismatch detection [7]–[9]. In both yeast and mammalian cells, there are three MutL complexes: MutLα, MutLβ, and MutLγ [7]–[9]. Downstream processing usually involves MutLα, in yeast a heterodimer of Mlh1 and Pms1 [7]–[9]. In yeast, it appears that both MutLβ (consisting of Mlh1 and Mlh2) and MutLγ (Mlh1 and Mlh3) play a role in correction of deletion mutations, although the effect is minor and depends on a sensitive assay [20], [21]. Although MutL proteins are not thought to have any specific recognition of mismatches, two mutations in *PMS1*, *pms1-G882E* and *pms1-H888R*, were shown to result in substantial increases in +1 insertions but had essentially no effect on repair of base-base mismatches or deletions [22].

Biochemical analysis has given no information about how MMR could differentiate between mismatches that would lead to insertion versus deletions. The experiments above that have indicated that MMR might repair insertion and deletion loops differently have been rather limited, and we wished to examine in/del mutagenesis in an environment in which sequence context, transcriptional strand, and replication strand could be controlled. We had previously found that we could generate insertion mutations of various sizes and compositions *in vivo* via single-strand oligonucleotide (oligo) transformation that was subject to MMR [23]. In that case, the oligos produced loops on the primer strand of replication that in the absence of repair led to insertion mutations; we had not tested whether oligos could induce loops on the template strand of replication that would lead to deletion mutations. Here we show that, in the absence of MMR, oligo transformation can be used to induce template-strand loops that lead to deletion mutations (deletion loops) with essentially the same efficiency as primer-strand loops that lead to insertion mutations (insertion loops). Using this assay we find that, when only MutSα is present, insertion loops are repaired with a greater efficiency than deletion loops, whereas in the presence of only MutSβ, insertion loops are poorly repaired, but deletion loops are efficiently repaired. Deletion loops are repaired almost as efficiently in strains containing *pms1-G882E* or *pms1-H888R* as in wild type strains, whereas insertion loops are not repaired. Surprisingly, repair of deletion loops in *pms1-G882E* or *pms1-H888R* mutant strains has a major dependence on MutLγ. Our data indicate that the biased repair of insertion versus deletion loops is dependent on interactions with the MutL proteins. We suggest that these properties of MMR can best be understood in an evolutionary sense in which MutSα represents the functions of bacterial MutS to repair base-base mismatches and in/del mismatches with a bias toward insertions, whereas MutSβ represents a new function present in some eukaryotes that complements MutSα function with respect to repair of deletion mismatches, chiefly through a different interaction with MutL proteins.

## Results

### An assay for in/del mutations

**Figure 1:**
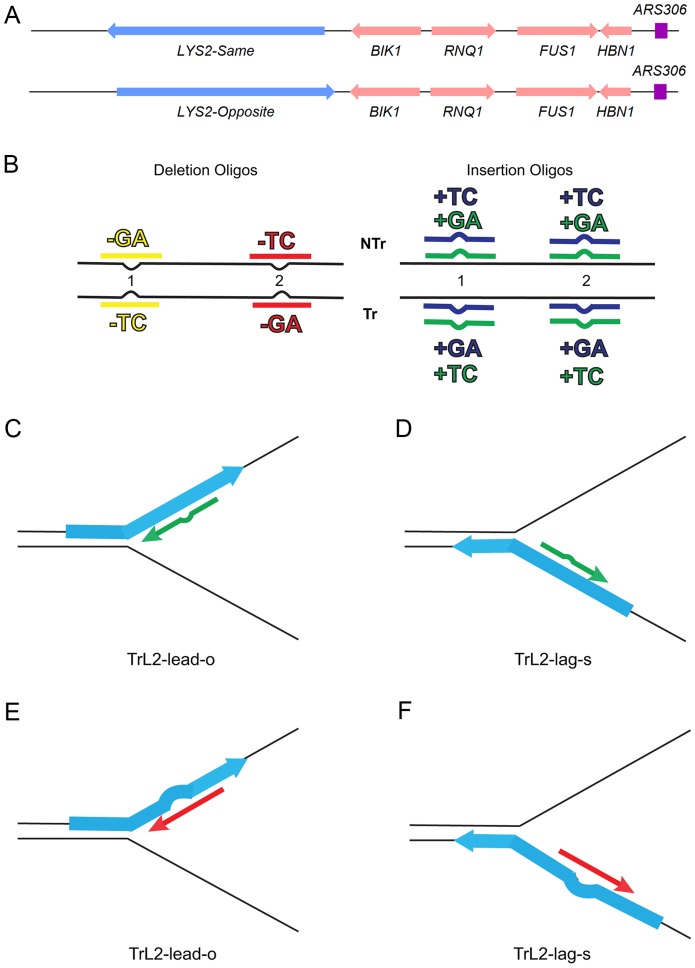
An assay for loop repair. (A) The initial strains used to construct the assay for in/del loop repair were a set of isogenic strains containing the *LYS2* gene replacing the *HIS4* gene near the *ARS306* origin of replication as shown above [26]. “Same” and “Opposite” refer to the orientation of the *LYS2* gene relative to the orientation of the original *HIS4* gene [26]. The wild-type *LYS2* sequences were subsequently replaced with sequences to create either the −1 frameshift allele *lys2ΔA746* or the +1 frameshift allele *lys2ΔBgl* [23]–[25]. (B) The −1 *lys2ΔA746* and the +1 *lys2ΔBgl* frameshift alleles can be reverted to wild-type by a compensating addition or deletion of nucleotides anywhere within an approximately 200-bp reversion window [23]–[25]. Oligos with sequences corresponding to two different locations within the reversion window of the mutant alleles and ranging in size from 31–36 nt were used to produce Lys+ revertants (Table S4). The colors indicated are those used in subsequent figures and also in Table S4. The red and yellow oligos induce a 2-nt loss in *lys2ΔA746* strains and the blue and green oligos insert 2 nt into *lys2ΔBgl* strains. Single-stranded oligos are used for transformation, and can therefore have the sequence of either the transcribed (Tr) or non-transcribed strand (NTr). Oligos inducing 1-nt in/del mutations follow a similar color and naming scheme (see text for details). (C–F) Oligos transform by serving as primers for subsequent replication, on either the leading or lagging strands of replication. If the mismatch created by the oligo is not removed during replication, a reverting frameshift will result in the next round of replication. Additional nucleotides in the oligo will create a primer-strand loop and thus an insertion mutation; missing nucleotides in the oligo will create a loop on the template strand and thus lead to a deletion mutation. (C) and (D) indicate that the same oligo (an oligo with the sequence of the transcribed strand (Tr) in location 2 (L2) adding a sequence of TC) will anneal to the leading strand of replication in *lys2ΔBgl* strains of the Opposite orientation (TrL2-lead-o) or to the lagging strand in strains of the Same orientation (TrL2-lag-s). (E) and (F) show the same process for an oligo inducing a deletion of GA in *lys2ΔA746* strains by annealing on the leading strand in strains of the Opposite orientation (TrL2-lead-o) or on the lagging strand of strains in the Same orientation (TrL2-lag-s).

We had previously used oligo transformation to study insertion mutations using the *lys2ΔA746* frameshift reversion assay that requires restoration of a −1 frameshift in a region of the *LYS2* gene indifferent to amino acid sequence [23], [24]. We wanted to study the effects of MMR on deletion mutations as well as insertion mutations, but, because of the different affinities in binding of loop sizes by MutSα and MutSβ, it was necessary to compare the effects of insertion and deletion mismatches of the same size, requiring the use of two complementary reversion assays. We therefore used both the −1 *lys2ΔA746* frameshift allele and the +1 frameshift allele *lys2ΔBgl* in the same *LYS2* region [23]–[25]. In order to have reversion windows with known orientations relative to a dependable origin of replication and to have the different frameshift alleles as similar as possible, we used the *LYS2* genes inserted in both orientations (“same” and “opposite”) at the *HIS4* locus previously described [26] and inserted the frameshift alleles as described in MATERIALS AND METHODS. The −1 *lys2ΔA746* frameshift allele was used to study +1 and −2 loops, and the +1 frameshift allele *lys2ΔBgl* was used to study −1 and +2 loops. The overall scheme for the assay is illustrated in Figure 1.

The efficiency of recognition by MMR is known to be dependent not only on the mispaired bases, but also on the sequence context surrounding the mispaired bases [10], [27]. Therefore we used a collection of oligos that created different mispairs, in two sequence contexts (Figure 1B). Because we transform with single-stranded oligos, the oligos can have the sequence of the transcribed strand and create a TC or GA insertion loop or have the sequence of the nontranscribed strand and create a TC or GA insertion loop, all in otherwise the same sequence context. This was done in two different locations within the reversion windows of the *lys2* mutant alleles. Deletion loops are created by transforming with oligos lacking certain bases contained in the template strand; therefore different deletion loop sequences cannot be created in the same sequence context using the same strains.

### Oligos induce 2-nt insertion or deletion mutations with approximately the same efficiency in the absence of MMR

**Figure 2:**
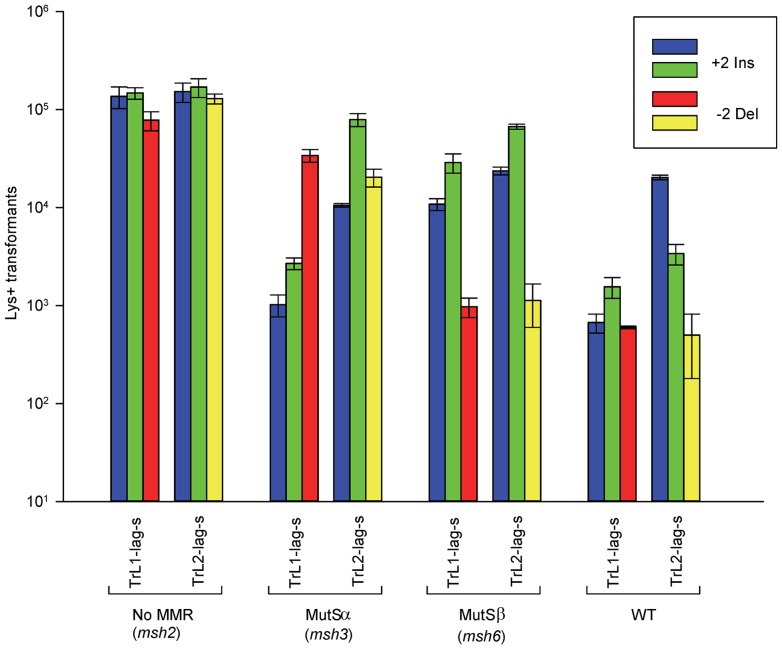
Effect of MMR on 2-nt in/del mismatches. The mean number of Lys+ revertants, with standard deviation, is shown for the indicated oligo and strain combination. The coloring is explained in Figure 1 and oligo sequences are given in Table S4. TrL1 and TrL2 refer to oligos with the sequence of the transcribed strand in Location 1 and 2, respectively. For the Tr oligos, annealing to the lagging strand occurs in strains with the Same orientation (Lag-s). The fewer transformants obtained for a given oligo and strain combination, the better the repair for the mismatch created by the oligo. Oligos creating insertion loops are transformed into *lys2ΔBgl* strains and oligos creating deletion loops are transformed into *lys2ΔA746* strains. As an example, all TrL1 oligos are essentially identical in sequence, with the exception that the “blue” oligo inserts a +GA loop, the “green” oligo inserts a +TC loop, and the “red” oligo causes a 2-nt −GA deletion loop in the template strand opposite the location of the + loops in the other two oligos. There is no active MMR in *msh2* strains, whereas *msh3* strains have MutSα present and *msh6* strains contain MutSβ.

As detailed in Materials and Methods, an oligo was transformed into a given strain in three independent experiments, and the average number of transformants over background reversion events was determined. All oligos were transformed into two strains with opposite orientations of the *LYS2* gene relative to the nearby origin of replication so that the effect of loops on the leading versus lagging strand could be assessed. The results of transformation with a selected set of oligos in strains containing or lacking certain components of MMR are given in Figure 2 and the full set of results is given in Figure S1.

Several patterns can be observed in the results in strains lacking MMR (*msh2*). Each pair of oligos differing only in the insertion bases gave results that were generally not statistically different from one another. Comparing any set of oligos (e.g. TrL1-Lag-s), the difference between insertions and deletions was generally less than 2-fold, with a mixture of insertions, deletions, or neither predominating. Finally, as we have observed previously [23], [28], in all cases the number of insertions or deletions was greater when targeted to the lagging strand than to the leading strand, by an average of approximately 6-fold in these experiments. Therefore we can conclude that, in the absence of MMR, insertion and deletion mutations can be created at approximately equal efficiencies by oligos.

### MutSα and MutSβ have opposite effects on 2-nt insertion versus deletion mispairs

In contrast to oligo transformation in the absence of MMR, one can see quite different patterns of transformation in strains containing only MutSα (*msh3* strains) or MutSβ (*msh6* strains) in Figure 2. To compare the effect of MMR on transformation, we divided the average number of revertants obtained in the absence of MMR by the average number of revertants obtained in a given MMR background to give a Repair Ratio (Table 1). The larger the Repair Ratio, the more effectively the loop created by the oligo was removed. The results in strains containing only MutSβ (*msh6* strains) are very consistent, as can be seen in Figures 2 and S1 and Table 1. In every case, deletion mispairs were corrected much more efficiently than insertion mispairs; in Table 1, the Repair Ratios for insertions range from 1 to 13 and for deletions from 59 to 310. The results in strains containing only MutSα (*msh3* strains) were more varied. Uniformly, deletion mispairs are poorly repaired, with a range of Repair Ratios of 2 to 9 (Table 1). Insertion mispairs are repaired with a wide range of efficiencies of 2 to 130 (Table 1). The one consistent difference is that within the same sequence context, a GA sequence in the loop is always repaired more efficiently than a TC. However, when only MutSα is present, insertion loops are repaired overall with much greater efficiency than deletion loops. Additionally, in the presence of only MutSα, insertion loops are repaired with somewhat greater efficiency when the loop is on the lagging strand compared to the leading strand, with an average ratio of 1.6, whereas, when only MutSβ is present, deletion loops on the leading strand are repaired 1.6-fold more efficiently than on the lagging strand, a difference we previously found under other circumstances [23]. The difference between these two ratios is statistically significant as determined by a Mann-Whitney rank sum test (P = 0.038). A median measure of the insertion and deletion loop Repair Ratios is given in Table 2, which illustrates the differing biases of MutSα and MutSβ.

**Table 1:** Repair Ratios for 2-nt in/del mispairs.

	Tr	NTr	*wt*	*msh3*	*msh6*	*pms1-H888R*	*pms1-H888R msh6*
**Location 1**							
**Lag-s**	+GA		200	130	13	1.1	
**Lag-o**		+TC	18	5	4	1.1	
**Lag-s**	+TC		95	55	5	1.0	
**Lag-o**		+GA	58	29	8	1.5	
**Lead-o**	+GA		58	55	6	1.3	
**Lead-s**		+TC	24	4	8	1.1	
**Lead-o**	+TC		32	22	3	2.0	
**Lead-s**		+GA	58	20	12	1.4	
**Location 2**							
**Lag-s**	+GA		7	14	6	0.9	
**Lag-o**		+TC	88	56	3	1.9	
**Lag-s**	+TC		50	2	3	0.9	
**Lag-o**		+GA	22	53	4	2.1	
**Lead-o**	+GA		4	9	7	1.5	
**Lead-s**		+TC	21	30	1	0.3	
**Lead-o**	+TC		15	2	3	2.0	
**Lead-s**		+GA	7	79	2	0.2	
**Location 1**							
**Lag-s**	−GA		130	2	80	68	56
**Lag-o**		−TC	270	8	110	460	210
**Lead-o**	−GA		170	3	95	77	50
**Lead-s**		−TC	360	5	310	60	72
**Location 2**							
**Lag-s**	−TC		260	6	120	130	120
**Lag-o**		−GA	130	9	59	180	140
**Lead-o**	−TC		280	6	96	180	110
**Lead-s**		−GA	160	4	100	72	75

Data from Figures S1 and S2 were used to calculate Repair Ratios by using the ratio of revertants obtained in the absence of MMR (*msh2* strains) with the number of revertants in strains of the indicated genotypes. Only MutSβ is present in *msh6* strains and only MutSα is present in *msh3* strains.

**Table 2:** Median Repair Ratios for 2-nt in/del mismatches.

	Median Repair Ratio		
MMR Genotype	Insertions	Deletions	Difference^a^
*msh3* (MutSα)	26	5.5	P = 0.018
*msh6* (MutSβ)	4.5	100	P = <0.001
wt	28	220	P = <0.001
*pms1(761-904)Δ*	1.8	2.4	N.S.
*pms1-G882E*	1.9	50	P = <0.001
*pms1-H888R*	1.2	100	P = <0.001
*mlh3*	180	54	P = 0.008
*pms1-G882E msh6*		42	
*pms1-H888R msh6*		95	
*pms1-H888R mlh3*		16	
*pms1-H888R msh6 mlh3*		16	

The median Repair Ratio for each genotype is calculated from the values with individual oligos in Tables 1, 5, and S1.

a The probability that the values for insertions were different from deletions was calculated using a Mann-Whitney rank sum test. N.S. indicates the two sets of values were not significantly different.

### Deletion mispairs of 2 nt are more efficiently corrected than insertion mispairs in wild-type strains

There is inherently more error associated with measurement of revertants in cells that are wild type for MMR, as the number of revertants can be decreased by over two orders of magnitude to quite low numbers. However, a consistent pattern emerges as observed both in Figures 2 and S1 and in Tables 1 and 2: 2-nt deletion mispairs are corrected more efficiently than insertion mispairs in strains wild type for MMR. Deletion mispairs are corrected with an efficiency somewhat greater than that of cells containing MutSβ alone (usually less than two-fold), presumably reflecting the ability of MutSα to recognize deletion mispairs, albeit at a much lower efficiency than does MutSβ. Insertion mispairs are generally corrected with an efficiency greater than that observed in cells with MutSα alone, although in 5 cases, insertion mispairs were corrected less efficiently than in MutSα cells, and in one other case about the same (Table 1). One explanation for those situations could be a dilution in MutSα molecules due to Msh3 pairing with some of the Msh2 [13], [14]. A dinucleotide repeat stability assay previously showed that 2-nt deletions were repaired with a greater efficiency than insertions in strains wild-type for MMR [18], [19].

### Two mutations in *PMS1*, *pms1-G882E* and *pms1-H888R*, result in repair deficiency of 2-nt insertions

**Figure 3:**
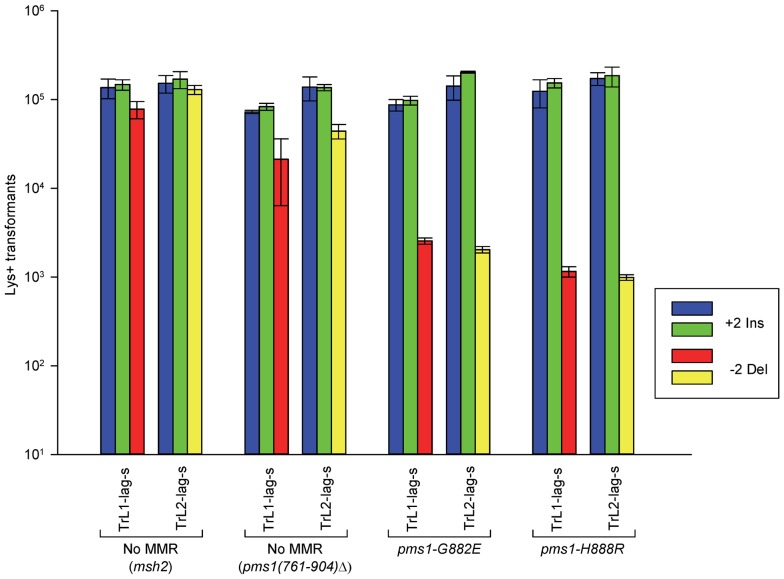
The effect of mutations in *PMS1* on 2-nt in/del mispairs. Oligos were transformed into strains of the indicated genotypes and analyzed as in Figure 2; the *msh2* results are those given in Figure 2.

A screen for mutations in *PMS1* found two mutants that resulted in large increases in +1 insertions but had no effect on deletions [22]. We tested those mutations in our assay system to see if they would have a similar effect on 2-nt in/del mispairs. The results are shown in Figures 3 and S2.

As described in Materials and Methods, the *pms1(761-904)Δ* mutant was a precursor in construction of the two *PMS1* point mutations; terminal deletions of that length have previously been shown to be nonfunctional [29]. Pms1 is needed for most repair, as the *pms1(761-904)Δ* strains behave similarly to the *msh2* strains. However, the *msh2* strains generally had more transformants, averaging 1.7-fold more insertions and 2.5-fold more deletions (Table S1), suggesting that some in/del repair might be mediated by complexes lacking Pms1. Strains containing either of the two *PMS1* point mutations show an extreme difference in repair of insertion versus deletion mispairs that is evident in Figures 3 and S2 and given quantitatively in Tables 1 and S1. Both mutant strains repair deletion mispairs but have little effect on insertion mispairs. The median effect of each mutation is presented in Table 2. The effect of the two mutations, *pms1-G882E* and *pms1-H888R* are similar, but the *pms1-H888R* mutants appear to have a more distinctive effect, with almost no repair of insertion mispairs but more repair of deletion mispairs than the *pms1-G882E* mutants. Because there is very little repair of deletion mispairs in the absence of Pms1 (Table S1), the *pms1* point mutants must be functional in deletion repair.

### Similar MMR effects are observed in 1-nt in/del mispairs

**Figure 4:**
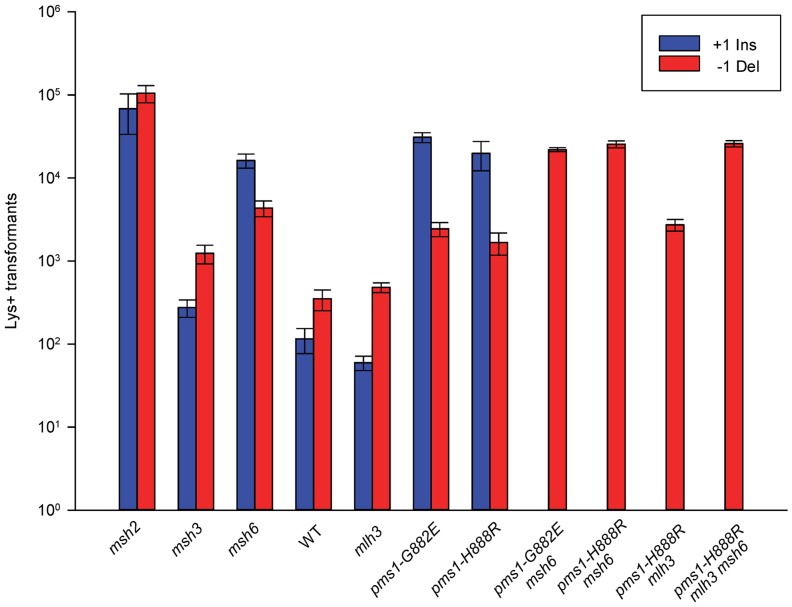
Effect of MMR on 1-nt in/del mismatches. TrL1 Oligos were transformed into Same-orientation strains of the indicated genotypes and analyzed as in Figure 2 (TrL1-Lag-s). For 1-nt in/del mismatches, oligos creating insertion loops are transformed into *lys2ΔA746* strains and oligos creating deletion loops are transformed into *lys2ΔBgl* strains. Only MutSβ is present in *msh6* strains and only MutSα is present in *msh3* strains.

Previously, the evidence for the differential effect of MutSα and MutSβ on in/del mutations came from a dinucleotide repeat assay, although an assay using one particular mononucleotide repeat indicated that the loss of either MutSα or MutSβ led to an increase mainly of deletions [18]. The *pms1-G882E* and *pms1-H888R* mutations had only been examined with mononucleotide repeats [22]. Therefore we wanted to examine whether the effects we observed on 2-nt in/del mispairs would be observed in similar 1-nt in/del mismatches. For that survey, we used only oligos in one location, and the results are presented in Figures 4 and S3; quantitative comparisons are given in Table 3.

**Table 3:** In/del Repair Ratios for 1 nt mispairs.

	Tr	NTr	*wt*	*msh3*	*msh6*	*pms1-H888R*	*mlh3*	*pms1-H888R mlh3*	*pms1-H888R msh6*	*pms1-H888R mlh3 msh6*
**Location 1**										
**Lag-s**	+T		590	250	4	3.4	990			
**Lag-o**		+T	240	110	2	0.8	170			
**Lead-o**	+T		120	210	4	1.1	970			
**Lead-s**		+T	210	170	2	4.1	730			
**Location 1**										
**Lag-s**	−T		300	85	24	63	220	38	4	4
**Lag-o**		−A	290	60	22	400	80	32	29	21
**Lead-o**	−T		360	29	13	91	87	1.4	5	29
**Lead-s**		−A	720	110	83	250	320	8	24	8

Data from Figure S3 were used to calculate Repair Ratios by using the ratio of revertants obtained in the absence of MMR (*msh2* strains) with the number of revertants in strains of the indicated genotype.

There are similarities to the results with 2-nt in/del mismatches in terms of the opposing biases for insertions versus deletions, but the quantitative results differ, presumably due to the relatively greater affinity of MutSα recognition for 1-nt loops over 2-nt loops, and the correspondingly lower recognition of MutSβ for 1-nt loops compared to 2-nt loops. MutSα has an overall much greater effect on suppression of 1-nt in/del mismatches than does MutSβ, and MutSα has substantial activity on 1-nt deletion loops in contrast to its activity on 2-nt deletion loops (Figures 4 and S3). Even so, MutSα has a consistently greater activity toward 1-nt insertion mismatches, whereas the MutSβ activity is the reverse. In contrast, the *pms1-G882E* and *pms1-H888R* mutants have about the same lack of insertion repair as exhibited on 2-nt in/del mispairs (Tables 3, S2). However, deletion repair in the *pms1-G882E* and *pms1-H888R* mutants is much more efficient than that in strains containing only MutSβ, indicating the involvement of MutSα in 1-nt deletion loop repair. The median Repair Ratios are given in Table 4 and illustrate that in contrast to the situation with 2-nt loops, there is relatively more repair of deletions with MutSα only and relatively less repair of deletions with MutSβ only, and in wild-type cells insertions and deletion mismatches are corrected with indistinguishable efficiency.

**Table 4:** Median Repair Ratios for 1-nt in/del mismatches.

	Median Repair Ratio		
MMR Genotype	Insertions	Deletions	Difference^a^
*msh3* (MutSα)	190	73	P = 0.017
*msh6* (MutSβ)	3	22	P = 0.029
wt	220	330	N.S.
*pms1-G882E*	2.3	86	P = 0.029
*pms1-H888R*	2.3	170	P = 0.029
*mlh3*	850	150	P = 0.029
*pms1-G882E msh6*		19	
*pms1-H888R msh6*		15	
*pms1-H888R mlh3*		20	
*pms1-H888R msh6 mlh3*		15	

The median Repair Ratio for each genotype is calculated from the values with individual oligos in Tables 3 and S2.

a The probability that the values for insertions were different from deletions was calculated using a Mann-Whitney rank sum test. N.S. indicates the two sets of values were not significantly different.

### The interaction of MutSα and *PMS1* mutations in in/del repair

**Figure 5:**
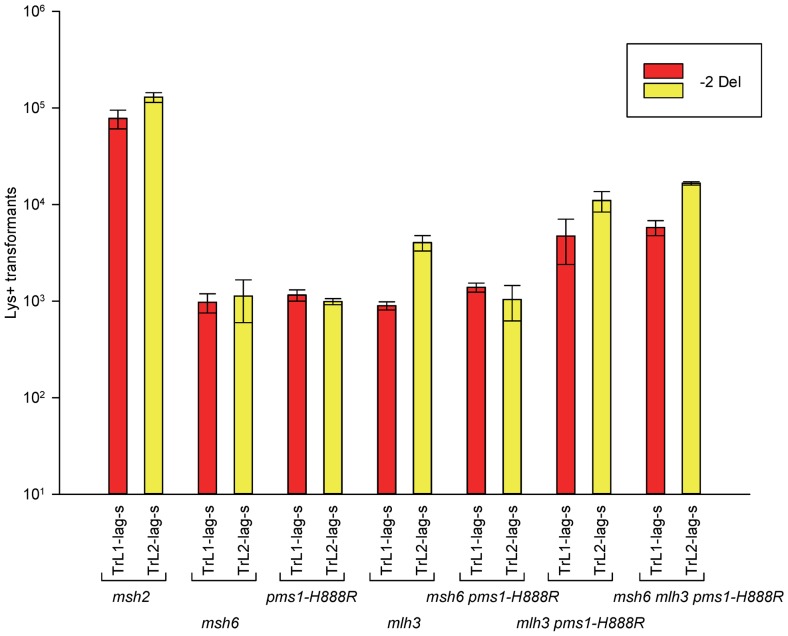
Effect of Mlh3 on 2-nt deletion mispairs. Oligos were transformed into strains of the indicated genotypes and analyzed as in Figure 2. (Data for *msh2*, *msh6*, and *pms1-H888R* are from Figures 2 and 3.)

How can the specificity of the *pms1-G882E* and *pms1-H888R* mutations best be understood? The *pms1-G882E* and *pms1-H888R* mutant strains appeared to be similar to *msh6* strains lacking MutSα for 2-nt deletion repair; we therefore examined strains containing both *msh6* deletions and *pms1* mutations to determine if they appeared to be in the same pathway. Because the *pms1* mutations fail to repair insertion loops, we could only examine the effect on deletion loop mispairs. The results are given in Figures 5 and S4 and Tables 1, 2, and S1 for 2-nt deletions. Results in the double mutants, *pms1-H888R msh6* and *pms1-G882E msh6*, are not distinguishable from the single *pms1* mutant results (Table 2).

For 1-nt deletion loop repair, MutSα is much more important than in 2-nt loop repair and as noted above, the repair in the *pms1* mutants is more efficient than in the presence of only MutSβ. Repair of 1-nt deletion loops in the double *pms1-G882E msh6* and *pms1-H888R msh6* mutants is much lower than in the single *pms1* mutants (Figure 4; Tables 3, S2), indicating that much of the deletion loop repair in the *pms1* mutants must be due to the action of MutSα (Table 4).

### The role of MutLγ in in/del repair

In order to determine if MutLγ (composed of Mlh1 and Mlh3 subunits [21]) might be involved in some of the observed repair, we examined strains with an *MLH3* deletion. The results for 2-nt in/del mispairs are given in Figures 5 and S4 and Tables 2 and 5. It is evident that MutLγ is not involved in repair of insertion mispairs, as Repair Ratios actually increased in the absence of MutLγ (P = 0.019) (perhaps due to a somewhat increased amount of MutLα). An *mlh3* deletion resulted in an approximately 4-fold decrease in repair of deletion mispairs (Table 2) (P = <0.001). Those results were expected given the limited effect previously found for *mlh3* deletions [10], [20], [21]. The *pms1-H888R* mutation has less than a 2-fold effect on deletion repair, so one would have anticipated that the double mutant would be similar to the *mlh3* mutant. Such was not the case as seen in Tables 2 and 5. The double mutant had an almost 13-fold reduction in deletion repair compared to wild type. The difference between repair in *mlh3* and *pms1-H888R mlh3* strains is significant, with P = <0.001.

**Table 5:** The effect of Mlh3 on Repair Ratios for 2-nt in/del mispairs.

	Tr	NTr	*mlh3*	Tr	NTr	*mlh3*	*pms1-H888R mlh3*	*pms1-H888R msh6 mlh3*
**Location 1**								
**Lag-s**	+TC		280	−GA		87	14	14
**Lag-o**		+GA	160		−TC	56	18	21
**Lead-o**	+TC		85	−GA		68	14	17
**Lead-s**		+GA	210		−TC	78	19	13
**Location 2**								
**Lag-s**	-	-	-	−TC		32	12	
**Lag-o**	-	-	-		−GA	51	18	
**Lead-o**	-	-	-	−TC		23	13	
**Lead-s**	-	-	-		−GA	51	24	

Repair Ratios were calculated as in previous tables.

The same pattern was found in 1-nt in/del mismatch repair. A single *mlh3* deletion has a relatively small effect on in/del repair, slightly raising the efficiency of insertion repair compared to wild type and slightly decreasing the efficiency of deletion repair, although the difference in both cases is marginally significant (P = 0.05) (Tables 3 and 4). The *pms1-H888R* mutant has robust deletion repair, but the double mutant *pms1-H888R mlh3* was reduced by 20 fold in deletion repair (the difference is significant, with P = 0.029); deleting *msh6* had no further effect (Tables 3 and 4). This result was particularly surprising, as MutSα is responsible for much of the 1-nt deletion repair and yet MutLγ has been thought to work only with MutSβ [21].

## Discussion

The biases we find here for repair of in/del mispairs had been previously observed in two different systems: a dinucleotide repeat assay for MutSα and MutSβ [18], [19] and frameshift reversion assays for the *pms1* mutants [22]. Given the limited scope of each of those experiments, it was not clear whether the results reflected a general property of the proteins involved, or were influenced by the DNA sequences involved in the particular assays used. Our results with a completely different assay system and with a variety of different sequences and gene strands and orientations lend confidence that our observations reflect an inherent difference in repair of insertion versus deletion loops by MMR.

For 2-nt in/del mismatches, strains containing only MutSβ provide the clearest picture of a bias. As shown in Table 1, the repair of all insertion loops tested is poor, ranging from 1 to 13-fold, and the repair of all tested deletion loops is robust, ranging from 60 to 300-fold. Although MutSβ has a measurable effect on repair of most insertions, it is only deletions for which it has a substantial effect. The effect in strains containing only MutSα is a bit more complex. The repair of deletion loops is uniformly low, ranging from 2 to 9-fold (Table 1). The repair of insertion loops is much more variable, with most (13/16) being repaired by a factor of 15 to 200-fold. The greater variability of repair initiated by MutSα compared to MutSβ is presumably a function of a stronger effect of sequence specificity [27]. The median Repair Ratios calculated in Table 2 indicate the remarkable difference between in/del repair mediated by MutSα and MutSβ although the individual data in Table 1 serve as a useful reminder that the particular repair of a given sequence can be quite variable.

Our analysis of repair of 1-nt in/del mispairs was not as extensive as that for 2-nt in/del mispairs, but the results we obtained reveal a similar pattern (Tables 3 and 4). Presumably due to the greater affinity for MutSα for 1-nt in/del mismatches compared to 2-nt in/del mismatches (and the converse for MutSβ) the overall effect of MutSα on repair of both insertion and deletion mismatches is much greater than for 2-nt in/del mismatches, and the effect of MutSβ for 1-nt deletion mismatches is much less than for 2-nt mismatches (Table 4 compared to Table 2). However, one can see that for all tested combinations, insertion loops are repaired more efficiently than deletion loops when only MutSα is present, and the reverse when only MutSβ is present. In this context, it is interesting to observe the overall effect on in/del mismatches as observed in strains wild type for MMR. For 2-nt in/del mismatches, one sees that on average deletion mispairs are repaired significantly better than insertion mispairs (Table 2), whereas for 1-nt mispairs, there is no consistent bias in repair (Table 4), reflecting the relatively greater effect of MutSα on repair.

How can the difference in repair of insertion versus deletion loops be explained? For that question, the existing evidence from biochemistry is not very helpful, as no biochemical experiments have been done in which the strands of duplex DNA that have been used could be identified as primer or template strands in a replication complex. Recent structural studies reveal that MutSβ binding to in/del mismatches takes place in a very different manner from MutSα or MutS binding to mismatches [30], [31]. Biochemical experiments have measured binding affinities, but what we measure here are overall repair efficiencies, for which the binding of MutSα or MutSβ is just the first step.

After MutSα or MutSβ binding, the next step in MMR is an association with a MutL protein complex, which is usually MutLα. If the repair efficiencies we measure were purely the result of MutSα or MutSβ binding efficiencies, then we would expect that any mutant of MutLα would have equivalent effects on in/del mismatch repair. However, the *PMS1* mutations we characterize, *pms1-G882E* and *pms1-H888R*, show extreme bias in in/del mismatch repair. As observed in Table 1, the effects of both mutations are approximately the same, with the *pms1-H888R* mutation showing a bit stronger effect. Strains with the *pms1-H888R* mutation show almost no repair of any insertion, but very robust repair of deletion mispairs, ranging from 60 to 460-fold. A similar effect is seen for 1-nt in/del mispairs (Table 3). The initial characterization of those two mutations showed that they had only a modest effect on overall mutation rate and that their mutator effect was primarily on frameshift deletions [22]. Our results with 2-nt in/del mispairs suggested that the *pms1-H888R* mutant behaved very much like strains containing only MutSβ, and the double-mutant strains did not seem to be significantly different from either of the single mutants (Table 2). However, as seen in Table 4, the situation is quite different with 1-nt in/del mispairs, as the double mutant strains are much worse at repair of deletions than either *pms1* mutant strain, indicating that much of the deletion repair in the *pms1-H888R* or *pms1-G882E* mutant is due to MutSα activity.

It is not clear how the two *pms1* mutants affect MMR. The two *pms1* mutations map into a region described as an Mlh1-interaction region [29], but the interaction of the mutant proteins with Mlh1 was not found to be defective as judged by a two-hybrid assay [22]. A recent structure of the *S. cerevisiae* MutLα C-terminal domain permits a much better understanding of the location of the mutations within the MutLα protein [32]. At the time of the Erdeniz *et al.* paper, the initiating ATG codon was thought to be in a location such that the length of the Pms1 protein would be 904 aa. However, a genomic analysis found a different ATG codon to be the correct initiation site for translation, leading to a predicted protein length of 873 aa [33]. With that numbering, the two Pms1 mutations would be G851E and H857R. The crystal structure shows that the H857 residue is centered in the β8 β-sheet that is part of one of the most important regions of the heterodimerization interface, Patch 1 [32]. The G851 residue sits just outside the β8 β-sheet and so it is reasonable to suppose that a mutation in that residue could affect Pms1-Mlh1 interaction. One of the zinc atoms in the endonuclease site is stabilized by C848 and H850 [32]. That would put the G851 residue close to the endonuclease site, making it possible that the G851E substitution might interfere with the binding of the zinc atom and thus affect endonuclease activity. However, there is no indication in the structure that the H857 residue would influence endonuclease activity, and as we found above, the H857R mutation has a more distinctive mutator effect than the G851E mutation. Both of the mutations were found to have essentially wild-type base-base MMR activity [22], and as Pms1 endonuclease activity is crucial for MMR function [34], we consider it highly unlikely that the effects of the two mutations is on the endonuclease activity of Pms1.

In accordance with previous results, we find that the absence of Mlh3 leads to somewhat less effective repair of deletion mispairs (Tables 2 and 4) [20], [21]. The repair of insertion mispairs in an *mlh3* background is more robust than in a wild-type background, suggesting that the loss of Mlh3 might lead to a somewhat greater amount of MutLα in the cell, with correspondingly greater repair of insertions. That view is consistent with the previous observation that overexpression of Mlh3 appears to result in lower levels of MutLα [35]. The surprise was the deletion repair observed in *pms-H888R mlh3* mutants. Given the small effect of each individual mutation on deletion repair, one would have expected deletion repair to be robust in that mutant background. Instead, repair of both 1-nt and 2-nt deletion mispairs was synergistically compromised (Tables 2 and 4). Based on the prior results with the *pms1-H888R* mutants, it appeared that only insertion repair was compromised [22]. Our results suggest a different possibility: although the *pms1-H888R* mutant is functional for base mismatch repair, it functions relatively poorly in in/del mismatch repair. One possible explanation for this hypothesis involves the finding that MutS complexes recognizing mismatches are responsible for loading multiple copies of MutLα onto DNA [36]. MutSα recognizing a base-base mispair can interact with the *pms1-H888R* mutant to create a functional complex. However because of the orientation of the proteins mediated by their binding to PCNA, neither MutSα nor MutSβ when recognizing an insertion mispair can interact properly with the *pms1-H888R* mutant complex and there is very little insertion repair. When MutSβ recognizes a deletion mispair, the complex is positioned so that it is able to interact with the *pms1-H888R* mutant MutLα, although relatively poorly, giving Repair Ratios of 16–20 (Tables 2 and 4). This interaction is facilitated by MutLγ interacting with MutSβ, which then helps recruit multiple molecules of the *pms1-H888R* mutant complex.

Repair of 2-nt deletion loops by MutSα is poor (Repair Ratio of 5.5, Table 2); however repair of 1-nt deletion loops by MutSα is much more robust (Repair Ratio of 73, Table 4), although still less than insertion loop repair. Repair of 1-nt deletion loops in the *pms1-H888R* mutant is much greater than repair with only MutSβ present (Repair Ratio of 170 compared to 22, Table 4), suggesting that much of the repair in the *pms1-H888R* mutant must be by MutSα. The fact that repair of 1-nt deletion loops in the *pms1-H888R mlh3* background drops to the level of repair when only MutSβ is present suggests that MutSα-directed repair in the presence of the *pms1-H888R* mutation involves MutLγ. The very modest effect of the *mlh3* mutation by itself shows that normal MutSα-directed repair of 1-nt deletion loops does not use MutLγ; confirmation of this suggestion would require additional experiments. One issue that has not been clear from previous experiments because of the modest effect of MutLγ on repair is whether there were certain mismatches that required MutLγ function, perhaps instead of MutLα, or whether the action of MutLγ always required MutLα and any mismatch was potentially susceptible to MutLγ function. Because each of our assays examines only one particular mismatch and because we see a strong effect in the *mlh3 pms1-H888R* background, we can draw several conclusions. 1) MutLγ functions only in repair of deletion loops and not insertion loops. 2) Any deletion loop is susceptible to being aided in repair by MutLγ. 3) MutLγ-mediated repair also requires MutLα. These conclusions do not mean that the effect of MutLγ deletion would be the same for all deletion loops: for both 1-nt and 2-nt deletion loops there is a range of about 4-fold in Repair Ratios, suggesting that certain mismatches could be more dependent for MutLγ on their repair.

The above model, while compatible with our results, makes several predictions that may however prove difficult to study. The first is that the bias in repair of insertions compared to deletions is ultimately a function of the MutL complexes and not the recognition by MutS complexes. A role for MutLγ in the repair of some deletion mispairs had previously been detected [20], [21], so the idea that MutL complexes could be biased in in/del repair is not without precedent. Secondly, the bias observed in in/del repair mediated by MutSα and MutSβ indicate that they contact MutLα differently such that a deletion mispair recognized by MutSβ is more likely to be repaired than if the same mispair were recognized by MutSα, and vice versa for insertion mispairs. A major question then is how the MutS and particularly the MutL components could be oriented such that an insertion mispair was recognized differently from a deletion mispair.

An important part of the explanation likely involves interactions of MMR proteins with the proliferating cell nuclear antigen, PCNA. PCNA is one of a family of DNA sliding clamps that encircles DNA, is essential for replication, and has binding sites for many proteins, including the replicative polymerases [37] and there is evidence that it can act as a scaffold to coordinate MMR through consecutive protein-protein interactions [38]. PCNA is required for MMR at a step preceding DNA resynthesis [39], [40], and MMR interactions with PCNA could be responsible for strand discrimination [41], [42]. A variety of experiments demonstrated direct interactions of PCNA with Mlh1, Msh3, and Msh6, and those interactions were important for proper MMR [40], [43]–[46]. It is clear that interaction with PCNA is not sufficient to drive MMR, as there are other processes occurring. For example, engineering a mutation that blocked MutSα conformational change upon mismatch binding demonstrated that such change was necessary for MutLα binding [47]. PCNA is asymmetrical with respect to the replication fork, and this asymmetry can result in specific MutLα loading and subsequent endonucleolytic activation and thus proper strand discrimination as has been observed in human MMR [42]. Importantly for this work, experiments with various PCNA mutants suggested that the interactions of PCNA are different for Msh3 compared to Msh6 [48]. In addition, it has been recently shown in humans that in contrast with MutSα, PCNA and MutLα have the same binding site on MutSβ, suggesting that the interaction of MutSβ with PCNA and MutLα would be sequential [49]. These considerations suggest a mechanism by which the recognition of, for example, an insertion loop could be different for MutSα compared to MutSβ because of their different orientation to the duplex bulge due to their different PCNA interaction. It is not clear how subsequent interactions with MutL complexes are handled. *In vitro* studies suggest that MutSα is bound to PCNA on homoduplex DNA, and, when a mispair is encountered, the interaction with PCNA is either lost or changed [50]. The next step of interaction with MutL complexes could be sequential for both MutS complexes, with a loss of the MutS interactions [38], but given the different nature of the MutS complex interactions with PCNA [49], the nature of the interactions of MutSα and MutSβ with MutLα is likely to be very different.

It is surprising to find that insertion and deletion mispairs are repaired with differing biases and that MutSα and MutSβ exhibit opposite biases for such repair. What might account for the development of an MMR system that would function in such a manner? A recent analysis was done of multiple strains of over 40 bacterial and archaeal species. It was found that in species with no MMR system, expansions and contractions of simple sequence repeats were equally likely, whereas in species containing MMR systems, there was a bias toward contraction of simple sequence repeats [51]. Thus, it appears that bacterial and archaeal MMR systems, like yeast strains containing only MutSα, repair insertions better than deletions. It is possible that such a bias could have an evolutionary advantage, tending to reduce the length of simple sequence repeats. Although most eukaryotic species seem to have an MMR system, not all have a MutSβ; in fact two favorite model organisms, *D. melanogaster* and *C. elegans*, lack MutSβ, although they both have MutSα [17]. Structural evidence also shows that MutSα binds mismatches in a manner similar to MutS, whereas MutSβ binds mismatches quite differently [30]. This analysis would suggest that MutSα represents the bacterial MutS activity, whereas MutSβ represents a new activity in which the bias toward repair of deletion mispairs may have been equally or more important than the recognition of larger loops. Many eukaryotic organisms have abundant simple sequence repeats, including those in exons, and the addition of a more robust activity repairing potential deletion mispairs would help preserve those repeats in the genome. This new MutSβ activity, due to the *MSH3* gene, not only had a recognition specificity different from that of MutSα, but interacted in a somewhat different manner with PCNA and MutLα and the new MutLγ complex that apparently does not usually interact with MutSα [21]. Domain swap experiments have shown that the mismatch recognition domain of Msh3 is not necessary for interaction with MutLγ, but rather another part of the Msh3 protein present in MutSβ [52].

Given the high degree of conservation, in both sequence and function, between MMR systems in yeast and mammalian cells, our results likely apply also to mammalian cells, although the experiments to test that are much more difficult to carry out. Repeat stability is a concern for mammalian cells, both in terms of various trinucleotide repeat diseases and in cancer [53], [54]. In various trinucleotide repeat diseases, there is a strong involvement with MMR, but the effects are complicated [53]. In a mouse model of Friedreich ataxia which has GAA repeats, repeat instability was increased in the absence of MMR and there were enhanced deletions in the absence of MutSβ and an enhancement of both deletions and insertions in the absence of MutSα, with a relatively greater increase in insertions [55]. Those results are consistent with the activities we report here. However, repeat instability of other types of trinucleotide repeats shows a different effect, with MMR appearing to be required for expansion, for example [53]. Although there is not a complete understanding of such effects, many of them involve MutSβ and interactions with larger loops. For example, there are certain types of loops that are repairable by MutSβ and others such as CAG loops in which the loop appears to maintain MutSβ binding, thus preventing repair [56]. However, in an *in vitro* assay, 1 or 2 repeats of CTG/CAG were repaired in a process requiring MutSβ, but not larger loops, or substrates that contained multiple loops on both strands [57].

Some of the first analyses of MMR genes in humans demonstrated that defects in MMR led to Lynch syndrome or hereditary nonpolyposis colorectal cancer and that such cells manifested a greatly enhanced microsatellite instability [1], [2]. Although the overall mutator effect of deficiencies in MMR is likely important in tumor formation and progression, genes containing exonic microsatellite sequences are a particularly susceptible target as any alteration in such sequences will likely lead to a strong phenotype [54], [58], [59]. Additionally there is some evidence that microsatellite repeats within introns and in 5′ and 3′ untranslated regions could also contribute to carcinogenesis [54]. Not only is the distribution of different tumor types generally different in MMR-defective mice compared to humans, but there is a marked difference depending on the particular defect in MMR [54], [60]. Our results provide additional information on possible reasons for those differences. Part of the difference between the distribution of tumor types in mouse and human is likely due to the difference in the existence and sequence of regions in cancer target genes susceptible to in/del formation. Although we are able to induce approximately equal frequencies of insertion or deletion mispairs in the absence of MMR, spontaneous formation of primer or template loops could be at least partially a function of sequence, sequence context, and replication on the leading versus lagging strand, thus also implicating the relation of the gene to replication origin. Because there is plasticity in use of replication origins, the same gene could be replicated differently depending on tissue type [61]. Not only could the formation of a loop be influenced by its sequence and location near an origin, but as we have demonstrated previously [23] and also find here, there is a bias in repair by MutSα and MutSβ depending on the replication strand. There is some variability with MutSβ with different oligos, but there is even more pronounced variability with MutSα, with almost a 100-fold difference in repair between the best- and worst-repaired oligo (Table 1). In both yeast and human cells, there seems to be generally more MutSα than MutSβ in cells, so the likelihood of repair of a given in/del will depend on how well it is recognized by MutSα or MutSβ, which could depend on a variety of factors including sequence and perhaps location, whether it is an insertion or deletion loop, and on which replication strand it appears on. If there turns out to be significant variability in the relative amounts of MutSα and MutSβ in various tissues, as has been found in mouse [16], the likelihood of repair could depend on tissue type. We demonstrate here the surprising finding that although the recognition of in/del mispairs is due to the MutS complex, it is the interaction with the MutL complex that biases the efficiency of repair of an insertion versus deletion mispair. Thus mutations in the genes encoding MutLα could influence not only the efficiency of repair but its bias in repair of in/del mispairs.

## Materials and Methods

### *S. cerevisiae* strains and oligos

The genotypes of strains used in these experiments can be found in Table S3. All strains were derivatives of SJR2259 and SJR22609 [26] with *LYS2* moved into *HIS4* location. Mutant *lys2* alleles either with [+1] (*lys2SΔBgl* and *lys2OΔBgl*) or [−1] (*lys2SΔA746* and *lys2OΔA746*) frameshifts were then introduced by two-step allele replacement [62] using plasmids pSR125 [63] or pSR786 [64] respectively. ‘S’ and ‘O’ refer to the orientation of the *LYS2* gene - the same or opposite orientation relatively to original *HIS4* orientation (Figure 1A). Gene deletions were made using a PCR fragment generated from the collection of yeast gene deletions [65]. The *pms1* point mutations were made using the delitto perfetto method [66]. The pCORE cassette was inserted into the *PMS1* gene using primers GCP735 and GCP736 (Table S4) creating the *pms1(761-904)Δ* mutant. The pCORE cassette was then replaced by transformation with a PCR product from strain NEY398 or NEY402 [22] using primers GCP737 and GCP738 (Table S4). Oligos for transformation were gel purified (Eurofins MWG Operon) and are listed in Table S4.

### Transformation with oligos

Transformation by electroporation was performed essentially as described previously [28], [67]. An overnight culture of yeast cells (0.5 ml) was inoculated into 25 ml of YPAD [68], incubated with shaking at 30° to an A_600_ of 1.3–1.5, washed twice with cold H_2_O, and once with cold 1 M sorbitol. After the final centrifugation, all solution was removed from the cells and 150 µL of cold 1 M sorbitol added to resuspend the cells. After addition of 200 pmol oligo and 50 ng of pRS314 [69] plasmid DNA, the solution was mixed and transferred into a 2-mm gap electroporation cuvette and electroporated at 1.55 kV, 200 Ω, and 25 uF (BTX Harvard Apparatus ECM 630). Immediately after electroporation, the cell suspension was added into 5 ml YPAD to recover for 2 h with shaking at 30°. Then cells were centrifuged, washed with H_2_O, and plated on synthetic dextrose (SD) medium lacking lysine [68]. The number of Trp+ transformants resulting from the pRS314 plasmid served as a useful marker of successful transformations, but was not consistent enough to be used as an internal standard for transformation efficiency. In order to determine background reversion, the same strains were electroporated as described but without adding oligos. For each oligo and strain combination, three independent experiments were performed, and the mean and standard deviation of the number of total transformants calculated.

## Supporting Information

Figure S1 Effect of MMR on 2-nt in/del mismatches. The mean number of Lys+ revertants, with standard deviation, is shown for each oligo and strain combination. The coloring is explained in Figure 1 and oligo sequences are given in Table S4. TrL1, TrL2, NTrL1, and NTrL2 refer to oligos with the sequence of the transcribed or nontranscribed strand in Location 1 and 2, respectively. For the Tr oligos, annealing to the lagging strand occurs in strains with the Same orientation (Lag-s), and to the leading strand in the Opposite orientation (Lead-o); the reverse is true for NTr oligos. Oligos creating insertion loops are transformed into *lys2ΔBgl* strains and oligos creating deletion loops are transformed into *lys2ΔA746* strains. As an example, all TrL1 oligos are identical in sequence, with the exception that the “blue” oligo inserts a +GA loop, the “green” oligo inserts a +TC loop, and the “red” oligo causes a 2-nt −GA deletion loop in the template strand opposite the location of the + loops in the other two oligos.
(TIF)

Figure S2 The effect of mutations in *PMS1* on 2-nt in/del mispairs. Oligos were transformed into strains of the indicated genotypes and analyzed as in Figure S1; the *msh2* results are those given in Figure S1.
(TIFF)

Figure S3 Effect of MMR on 1-nt in/del mismatches. Oligos were transformed into strains of the indicated genotypes and analyzed as in Figure 2. For 1-nt in/del mismatches, oligos creating insertion loops are transformed into *lys2ΔA746* strains and oligos creating deletion loops are transformed into *lys2ΔBgl* strains. Oligo sequences are given in Table S4. Only MutSβ is present in *msh6* strains and only MutSα is present in *msh3* strains.
(TIF)

Figure S4 Effect of Mlh3 on 2-nt deletion mispairs. Oligos were transformed into strains of the indicated genotypes and analyzed as in . (Data for *msh2*, *msh6*, and *pms1-H888R* from Figures S1 and S2.)
(TIF)

Table S1 Repair Ratios for 2 nt in/del mispairs.
(DOCX)

Table S2 Repair Ratios for 1 nt in/del mispairs in *pms1-G882E*.
(DOCX)

Table S3 *S. cerevisiae* strains.
(DOCX)

Table S4 Oligos used in this study.
(DOCX)
