# Supplementary figures and images for: Different Roles of Eukaryotic MutS and MutL Complexes in Repair of Small Insertion and Deletion Loops in Yeast
Source: PLoS Genet. 2013 Oct 31;9(10):e1003920. doi: 10.1371/journal.pgen.1003920 (PMC3814323; doi:10.1371/journal.pgen.1003920)

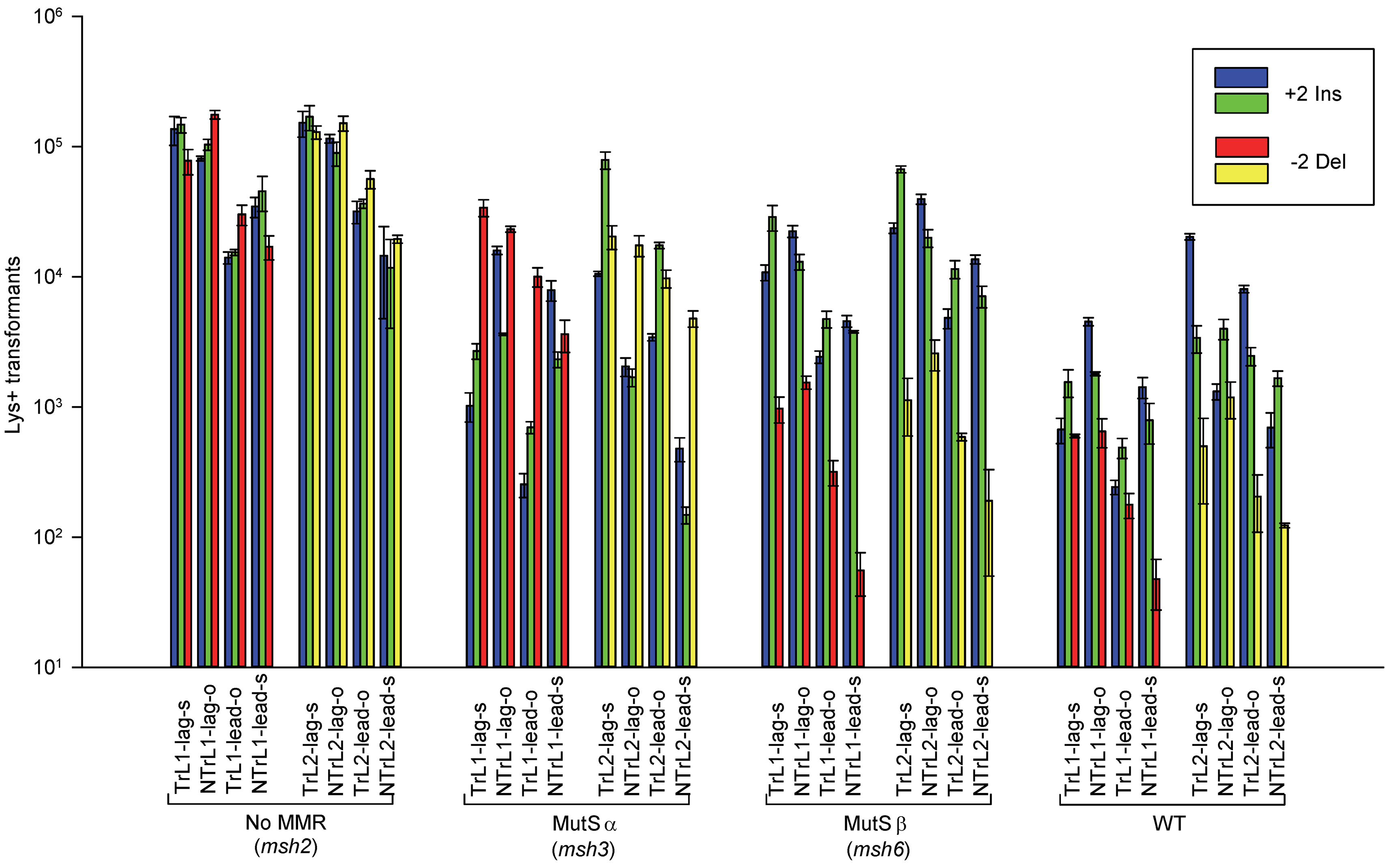

Supplement: Figure S1 — Effect of MMR on 2-nt in/del mismatches. The mean number of Lys+ revertants, with standard deviation, is shown for each oligo and strain combination. The coloring is explained in Figure 1 and oligo sequences are given in Table S4. TrL1, TrL2, NTrL1, and NTrL2 refer to oligos with the sequence of the transcribed or nontranscribed strand in Location 1 and 2, respectively. For the Tr oligos, annealing to the lagging strand occurs in strains with the Same orientation (Lag-s), and to the leading strand in the Opposite orientation (Lead-o); the reverse is true for NTr oligos. Oligos creating insertion loops are transformed into lys2ΔBgl strains and oligos creating deletion loops are transformed into lys2ΔA746 strains. As an example, all TrL1 oligos are identical in sequence, with the exception that the “blue” oligo inserts a +GA loop, the “green” oligo inserts a +TC loop, and the “red” oligo causes a 2-nt −GA deletion loop in the template strand opposite the location of the + loops in the other two oligos. (TIF) [file pgen.1003920.s001.tif]

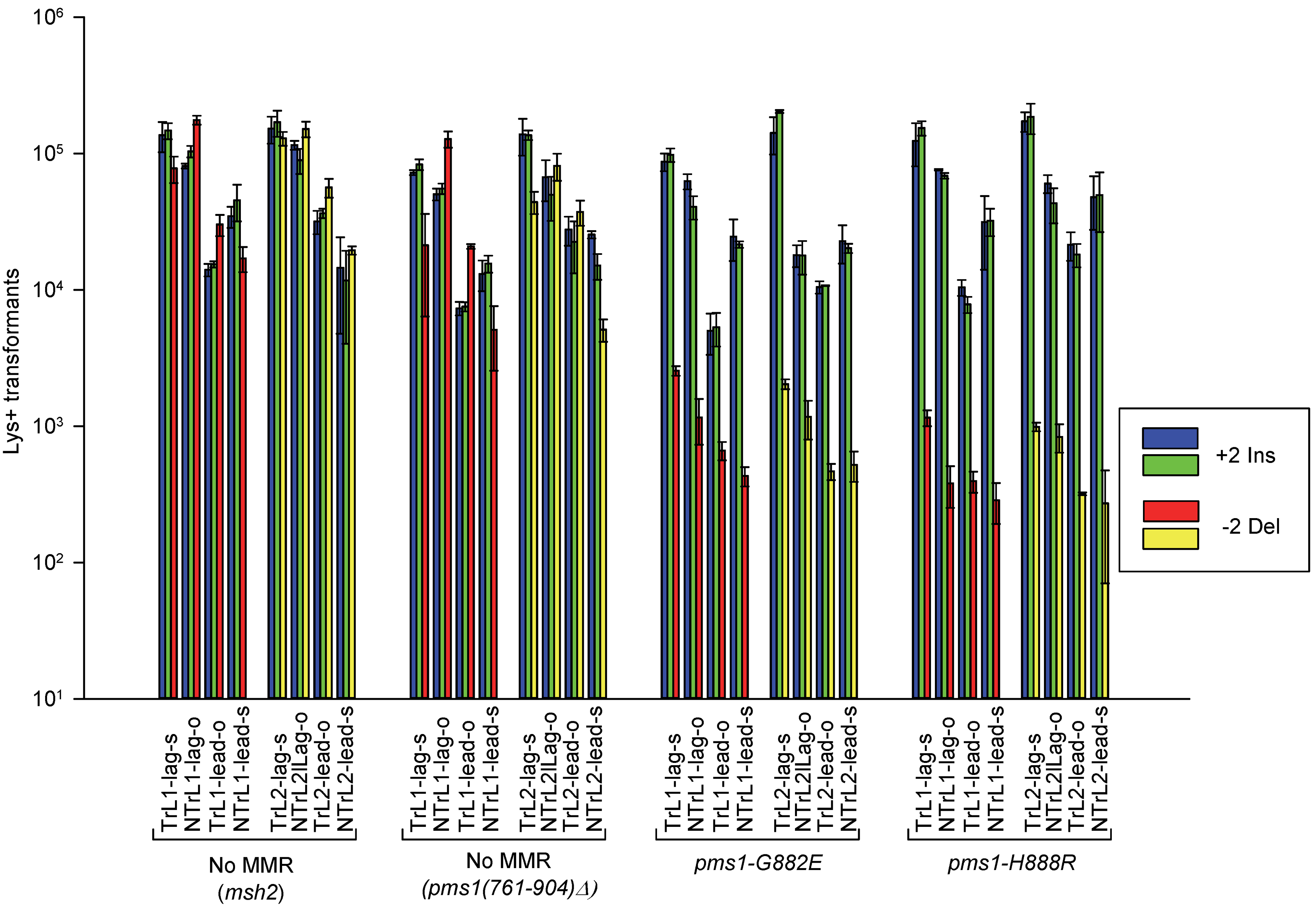

Supplement: Figure S2 — The effect of mutations in PMS1 on 2-nt in/del mispairs. Oligos were transformed into strains of the indicated genotypes and analyzed as in Figure S1; the msh2 results are those given in Figure S1. (TIFF) [file pgen.1003920.s002.tiff]

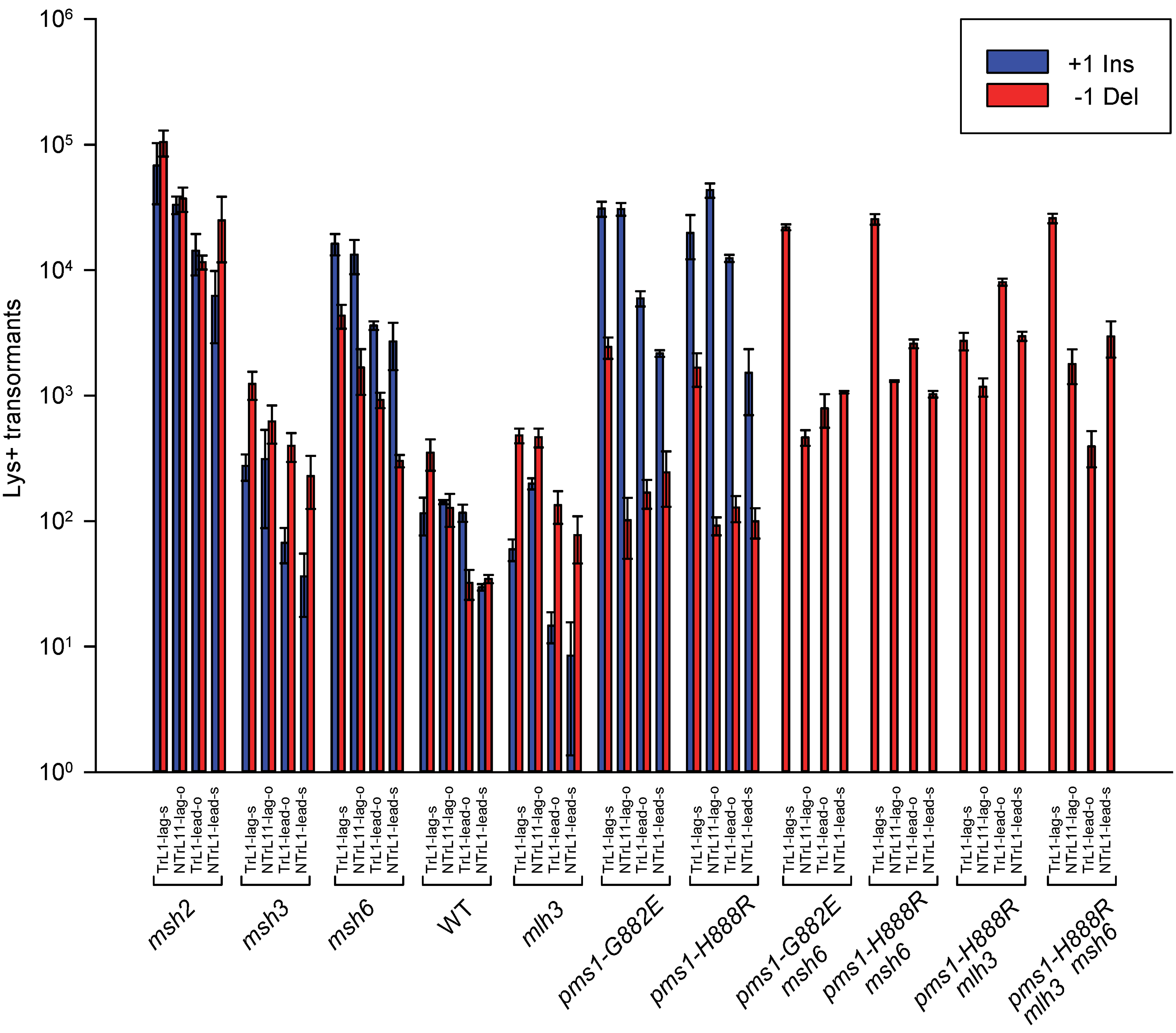

Supplement: Figure S3 — Effect of MMR on 1-nt in/del mismatches. Oligos were transformed into strains of the indicated genotypes and analyzed as in Figure 2. For 1-nt in/del mismatches, oligos creating insertion loops are transformed into lys2ΔA746 strains and oligos creating deletion loops are transformed into lys2ΔBgl strains. Oligo sequences are given in Table S4. Only MutSβ is present in msh6 strains and only MutSα is present in msh3 strains. (TIF) [file pgen.1003920.s003.tif]

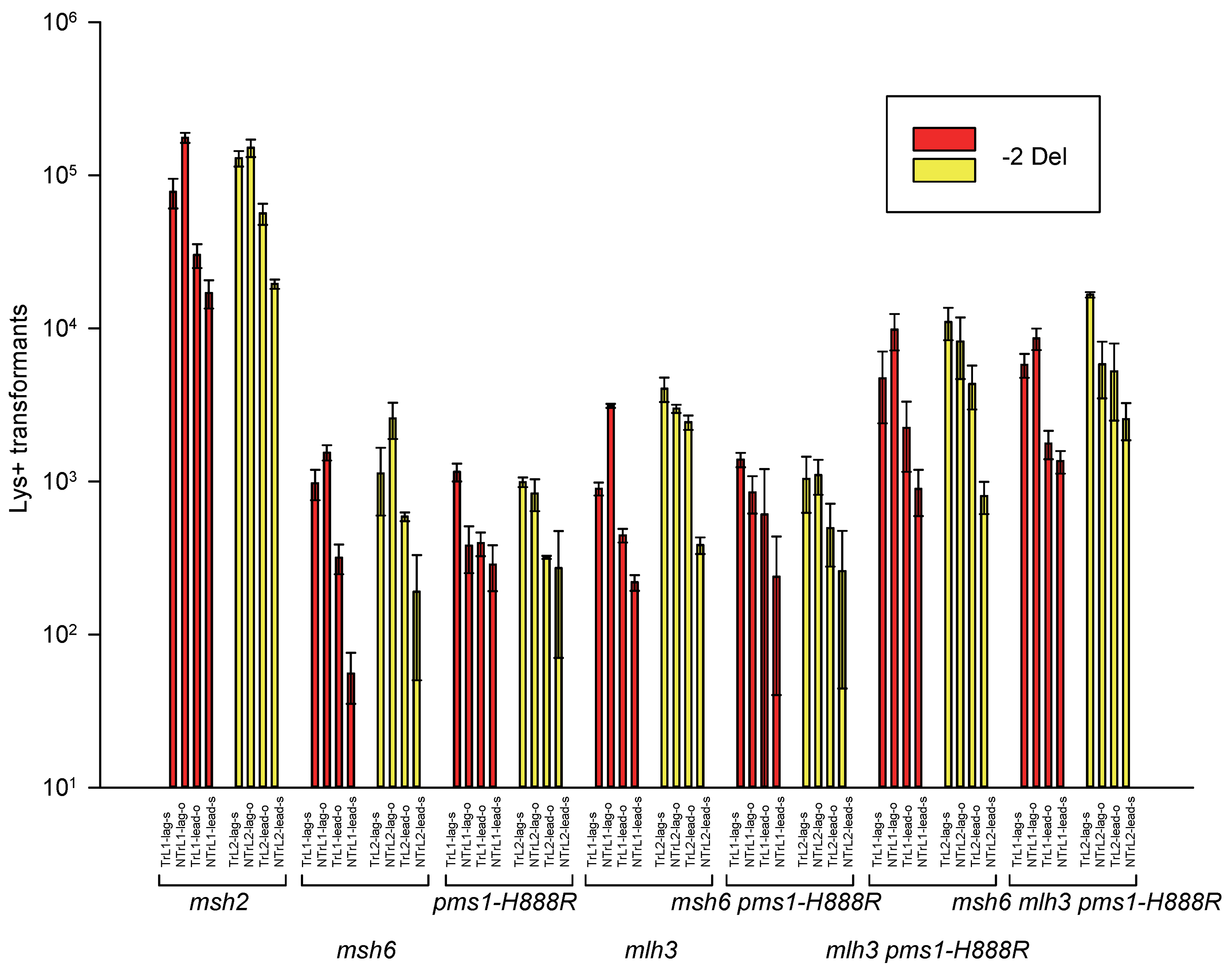

Supplement: Figure S4 — Effect of Mlh3 on 2-nt deletion mispairs. Oligos were transformed into strains of the indicated genotypes and analyzed as in . (Data for msh2, msh6, and pms1-H888R from Figures S1 and S2.) (TIF) [file pgen.1003920.s004.tif]
