# Supplementary material for: Different Roles of Eukaryotic MutS and MutL Complexes in Repair of Small Insertion and Deletion Loops in Yeast
Source: PLoS Genet. 2013 Oct 31;9(10):e1003920. doi: 10.1371/journal.pgen.1003920 (PMC3814323; doi:10.1371/journal.pgen.1003920)
Supplement: Table S1 — Repair Ratios for 2 nt in/del mispairs. (DOCX) [file pgen.1003920.s005.docx]

| **Table S1.** Repair Ratios for 2 nt in/del mispairs. | | | | | |
| --- | --- | --- | --- | --- | --- |
|  | **Tr** | **NTr** | ***pms1(761-904)Δ*** | ***pms1-G882E*** | ***pms1-G882E msh6*** |
| **Location 1** | |  |  |  |  |
| **Lag-s** | +GA |  | 1.9 | 1.6 |  |
| **Lag-o** |  | +TC | 1.6 | 1.3 |  |
| **Lag-s** | +TC |  | 1.8 | 1.5 |  |
| **Lag-o** |  | +GA | 1.9 | 2.5 |  |
| **Lead-o** | +GA |  | 1.9 | 2.8 |  |
| **Lead-s** |  | +TC | 2.6 | 1.4 |  |
| **Lead-o** | +TC |  | 2.0 | 2.9 |  |
| **Lead-s** |  | +GA | 2.9 | 2.1 |  |
| **Location 2** | |  |  |  |  |
| **Lag-s** | +GA |  | 1.1 | 1.1 |  |
| **Lag-o** |  | +TC | 1.7 | 6.4 |  |
| **Lag-s** | +TC |  | 1.2 | 0.8 |  |
| **Lag-o** |  | +GA | 1.8 | 5.0 |  |
| **Lead-o** | +GA |  | 1.1 | 3.0 |  |
| **Lead-s** |  | +TC | 0.6 | 0.6 |  |
| **Lead-o** | +TC |  | 1.6 | 3.4 |  |
| **Lead-s** |  | +GA | 0.8 | 0.6 |  |
| **Location 1** | |  |  |  |  |
| **Lag-s** | -GA |  | 3.7 | 31 | 24 |
| **Lag-o** |  | -TC | 1.4 | 150 | 90 |
| **Lead-o** | -GA |  | 1.4 | 46 | 25 |
| **Lead-s** |  | -TC | 3.4 | 40 | 45 |
| **Location 2** | |  |  |  |  |
| **Lag-s** | -TC |  | 2.9 | 90 | 49 |
| **Lag-o** |  | -GA | 1.9 | 54 | 41 |
| **Lead-o** | -TC |  | 1.5 | 140 | 42 |
| **Lead-s** |  | -GA | 3.8 | 10 | 31 |

Data from Figure S2 and other genotypes not shown were used to calculate Repair Ratios by using the ratio of revertants obtained in the absence of MMR (*msh2* strains) with the number of revertants in strains of the indicated genotype.
