# Supplementary material for: Different Roles of Eukaryotic MutS and MutL Complexes in Repair of Small Insertion and Deletion Loops in Yeast
Source: PLoS Genet. 2013 Oct 31;9(10):e1003920. doi: 10.1371/journal.pgen.1003920 (PMC3814323; doi:10.1371/journal.pgen.1003920)
Supplement: Table S2 — Repair Ratios for 1 nt in/del mispairs in pms1-G882E. (DOCX) [file pgen.1003920.s006.docx]

| Table S2. Repair Ratios for 1 nt in/del mispairs in *pms1-G882E.* | | | | | |  |
| --- | --- | --- | --- | --- | --- | --- |
|  | Tr | | NTr | *pms1-G882E* | *pms1-G882E msh6* | |
| Location 1 |  |  | |  |  | |
| Lag-s | +A |  | | 2.2 |  | |
| Lag-o |  | +T | | 1.1 |  | |
| Lead-o | +A |  | | 2.4 |  | |
| Lead-s |  | +T | | 2.9 |  | |
| Location 1 |  |  | |  |  | |
| Lag-s | -T |  | | 43 | 5 | |
| Lag-o |  | -A | | 370 | 80 | |
| Lead-o | -T |  | | 69 | 15 | |
| Lead-s |  | -A | | 100 | 23 | |
| Data from Figure 4 were used to calculate repair factors as in Table S1. | | | | | | |
