# Supplementary material for: Different Roles of Eukaryotic MutS and MutL Complexes in Repair of Small Insertion and Deletion Loops in Yeast
Source: PLoS Genet. 2013 Oct 31;9(10):e1003920. doi: 10.1371/journal.pgen.1003920 (PMC3814323; doi:10.1371/journal.pgen.1003920)
Supplement: Table S3 — S. cerevisiae strains. (DOCX) [file pgen.1003920.s007.docx]

| **Table S3.** *S.cerevisiae* strains | | |
| --- | --- | --- |
| **Strain** | **Genotype** | **Reference** |
| SJR2259 | WT *MATa ura3-52* *trp1Δ1* *lys2Δ::hyg* *leu2-K:TetR’-Ssn6::LEU2* [pSR857 integrated at *LEU2*] *his4Δ::LYS2* Same | [1] |
| SJR2260 | WT *MATa ura3-52* *trp1Δ1* *lys2Δ::hyg* *leu2-K:TetR’-Ssn6::LEU2* [pSR857 integrated at *LEU2*] *his4Δ::LYS2* Opposite | [1] |
| GCY2467 | WT SJR2259 *lys2SΔBgl* | This study |
| GCY2499 | *msh2::nat lys2SΔBgl* | This study |
| GCY2470 | *msh3::kan lys2SΔBgl* | This study |
| GCY2471 | *msh6::kan lys2SΔBgl* | This study |
| GCY2466 | WT SJR2259 *lys2SΔA746* | This study |
| GCY2498 | *msh2::nat lys2SΔA746* | This study |
| GCY2468 | *msh3::kan lys2SΔA746* | This study |
| GCY2469 | *msh6::kan lys2SΔA746* | This study |
| GCY2448 | WT SJR2260 *lys2OΔBgl* | This study |
| GCY2452 | *msh2::nat lys2OΔBgl* | This study |
| GCY2473 | *msh3::kan lys2OΔBgl* | This study |
| GCY2454 | *msh6::kan lys2OΔBgl* | This study |
| GCY2449 | WT SJR2260 *lys2OΔA746* | This study |
| GCY2453 | *msh2::nat lys2OΔA746* | This study |
| GCY2474 | *msh3::kan lys2OΔA746* | This study |
| GCY2455 | *msh6::kan lys2OΔA746* | This study |
| GCY2759 | *pms1* *(761-904)Δ lys2SΔA746* | This study |
| GCY2760 | *pms1* *(761-904)Δ lys2OΔA746* | This study |
| GCY2761 | *pms1* *(761-904)Δ lys2SΔBgl* | This study |
| GCY2762 | *pms1* *(761-904)Δ lys2OΔBgl* | This study |
| GCY2763 | *pms1G882E lys2SΔA746* | This study |
| GCY2764 | *pms1H888R lys2SΔA746* | This study |
| GCY2765 | *pms1G882E lys2OΔA746* | This study |
| GCY2766 | *pms1H888R lys2OΔA746* | This study |
| GCY2767 | *pms1G882E lys2SΔBgl* | This study |
| GCY2768 | *pms1H888R lys2SΔBgl* | This study |
| GCY2769 | *pms1G882E lys2OΔBgl* | This study |
| GCY2770 | *pms1H888R lys2OΔBgl* | This study |
| GCY2771 | *msh6::kan pms1-G882E lys2SΔA746* | This study |
| GCY2772 | *msh6::kan pms1-H888R lys2SΔA746* | This study |
| GCY2773 | *msh6::kan pms-1G882E lys2OΔA746* | This study |
| GCY2774 | *msh6::kan pms1-H888R lys2OΔA746* | This study |
| GCY2775 | *msh6::kan pms1-G882E lys2SΔBgl* | This study |
| GCY2776 | *msh6::kan pms1-H888R lys2SΔBgl* | This study |
| GCY2777 | *msh6::kan pms1-G882E lys2OΔBgl* | This study |
| GCY2778 | *msh6::kan pms1-H888R lys2OΔBgl* | This study |
| GCY2795 | *mlh3::kan pms1-H888R lys2SΔA746* | This study |
| GCY2796 | *mlh3::kan pms1-H888R lys2OΔA746* | This study |
| GCY2821 | *mlh3::kan pms1-H888R lys2SΔBgl* | This study |
| GCY2822 | *mlh3::kan pms1-H888R lys2OΔBgl* | This study |
| GCY2823 | *mlh3::nat msh6::kan pms1-H888R lys2SΔA746* | This study |
| GCY2824 | *mlh3::nat msh6::kan pms1-H888R lys2OΔA746* | This study |
| GCY2825 | *mlh3::nat msh6::kan pms1-H888R lys2SΔBgl* | This study |
| GCY2826 | *mlh3::nat msh6::kan pms1-H888R lys2OΔBgl* | This study |
| GCY2827 | *mlh3::kan lys2SΔA746* | This study |
| GCY2828 | *mlh3::kan lys2OΔA746* | This study |
| GCY2829 | *mlh3::kan lys2SΔBgl* | This study |
| GCY2830 | *mlh3::kan lys2OΔBgl* | This study |

1. Kim N, Abdulovic AL, Gealy R, Lippert MJ, Jinks-Robertson S (2007) Transcription-associated mutagenesis in yeast is directly proportional to the level of gene expression and influenced by the direction of DNA replication. DNA Repair (Amst) 6: 1285-1296.
