# Supplementary material for: Different Roles of Eukaryotic MutS and MutL Complexes in Repair of Small Insertion and Deletion Loops in Yeast
Source: PLoS Genet. 2013 Oct 31;9(10):e1003920. doi: 10.1371/journal.pgen.1003920 (PMC3814323; doi:10.1371/journal.pgen.1003920)
Supplement: Table S4 — Oligos used in this study. (DOCX) [file pgen.1003920.s008.docx]

| **Table S4.** Oligos used in this study. | | | |
| --- | --- | --- | --- |
| Oligos | Location | Allele transformed | Sequence |
| **2 nt Del** | **Location 1** |  |  |
| GCP719 | NTr1(-TC) | *lys2ΔA746* | ACCAAGATTTCAAATT--CGAGCTCAAGCATCATTT |
| GCP720 | Tr1(-GA) | *lys2ΔA746* | AAATGATGCTTGAGCTCG--AATTTGAAATCTTGGT |
| **2nt Del** | **Location 2** |  |  |
| GCP721 | NTr2(-GA) | *lys2ΔA746* | ATCTGGAAAGGACCCC--AGTTGTTCCGTTTGGCCT |
| GCP722 | Tr2(-TC) | *lys2ΔA746* | AGGCCAAACGGAACAACT--GGGGTCCTTTCCAGAT |
| **2 nt Ins** | **Location 1** |  |  |
| GCP723 | NTr1(+TC) | *lys2ΔBgl* | ACCAAGATTTCAAATTTCAGACGAGTTCAAGCATCA |
| GCP724 | Tr1(+GA) | *lys2ΔBgl* | TGATGCTTGAACTCGTCTGAAATTTGAAATCTTGGT |
| GCP725 | NTr1(+GA) | *lys2ΔBgl* | ACCAAGATTTCAAATTGAAGACGAGTTCAAGCATCA |
| GCP726 | Tr1(+TC) | *lys2ΔBgl* | TGATGCTTGAACTCGTCTTCAATTTGAAATCTTGGT |
| **2nt Ins** | **Location 2** |  |  |
| GCP727 | NTr2(+TC) | *lys2ΔBgl* | ATCTGGAAAGGACCCCTCTCAGTTGTTCCGTTTGGC |
| GCP728 | Tr2(+GA) | *lys2ΔBgl* | GCCAAACGGAACAACTGAGAGGGGTCCTTTCCAGAT |
| GCP729 | NTr2(+GA) | *lys2ΔBgl* | ATCTGGAAAGGACCCCGATCAGTTGTTCCGTTTGGC |
| GCP730 | Tr2(+TC) | *lys2ΔBgl* | GCCAAACGGAACAACTGATCGGGGTCCTTTCCAGAT |
| **1nt Del** | **Location 1** |  |  |
| GCP731 | NTr1(-A) | *lys2ΔBgl* | ACCAAGATTTCAAATT-GACGAGTTCAAGCATCA |
| GCP732 | Tr1(-T) | *lys2ΔBgl* | TGATGCTTGAACTCGTCT-ATTTGAAATCTTGGT |
| **1nt Ins** | **Location 1** |  |  |
| GCP733 | NTr1(+T) | *lys2ΔA746* | ACCAAGATTTCAAATTTGACGAGCTCAAGCATCA |
| GCP734 | Tr1(+T) | *lys2ΔA746* | TGATGCTTGAGCTCGTCATATTTGAAATCTTGGT |
|  |  |  |  |
| GCP735 | PMS1pCOREF |  | CTATGACCAGAGTCGTTCATAATCTCAGTGAACTTGATAAG  CCTTGGAATTGTCCCCATGAGCTCGTTTTCGACACTGG |
| GCP736 | PMS1pCORER |  | AAGCTAGATCATATTTCGTAATCCTTCGAAAATGAGCTCCAA  TCACGTAATTCCATTAATCCTTACCATTAAGTTGATC |
| GCP737 | Pms1MutF |  | GATAATACCTCAGCCGGTAG |
| GCP738 | Pms1MutR |  | CGTCCCTTTGGTCTTGTATC |
| The colors in the location are keyed to the figures. Because of the way in which the reversion windows in the *lys2ΔA746* and *lys2ΔBgl* alleles were constructed, the sequences corresponding to Location 1 differ in the two alleles in two positions, necessitating slight differences between the Ins and Del primers for Location 1. Nucleotides added by the oligos are underlined; dashes indicate the location of induced deletions. Where appropriate, the *lys2* allele used as a target for the oligo is indicated. | | | |
